# AKAP12 Mediates Barrier Functions of Fibrotic Scars during CNS Repair

**DOI:** 10.1371/journal.pone.0094695

**Published:** 2014-04-23

**Authors:** Jong-Ho Cha, Hee-Jun Wee, Ji Hae Seo, Bum Ju Ahn, Ji-Hyeon Park, Jun-Mo Yang, Sae-Won Lee, Eun Hee Kim, Ok-Hee Lee, Ji Hoe Heo, Hyo-Jong Lee, Irwin H. Gelman, Ken Arai, Eng H. Lo, Kyu-Won Kim

**Affiliations:** 1 SNU-Harvard NeuroVascular Protection Research Center, College of Pharmacy and Research Institute of Pharmaceutical Sciences, Seoul National University, Seoul, Korea; 2 Department of Molecular Medicine and Biopharmaceutical Sciences, Graduate School of Convergence Science and Technology, and College of Medicine or College of Pharmacy, Seoul National University, Seoul, Korea; 3 Department of Internal Medicine, and Innovative Research Institute for Cell Therapy, Seoul National University Hospital, Seoul, Korea; 4 Severance Biomedical Science Institute, Yonsei University College of Medicine, Seoul, Korea; 5 Severance Integrative Research Institute for Cerebral & Cardiovascular Disease, Yonsei University College of Medicine, Seoul, Korea; 6 Department of Neurology, Yonsei University College of Medicine, Seoul, Korea; 7 College of Pharmacy, Inje University, Gimhae, Korea; 8 Department of Cancer Genetics, Roswell Park Cancer Institute, Buffalo, New York, United States of America; 9 Neuroprotection Research Laboratory, Departments of Radiology and Neurology, Massachusetts General Hospital, and Harvard Medical School, Boston, Massachusetts, United States of America; Emory University School of Medicine, United States of America

## Abstract

The repair process after CNS injury shows a well-organized cascade of three distinct stages: inflammation, new tissue formation, and remodeling. In the new tissue formation stage, various cells migrate and form the fibrotic scar surrounding the lesion site. The fibrotic scar is known as an obstacle for axonal regeneration in the remodeling stage. However, the role of the fibrotic scar in the new tissue formation stage remains largely unknown. We found that the number of A-kinase anchoring protein 12 (AKAP12)-positive cells in the fibrotic scar was increased over time, and the cells formed a structure which traps various immune cells. Furthermore, the AKAP12-positive cells strongly express junction proteins which enable the structure to function as a physical barrier. In *in vivo* validation, AKAP12 knock-out (KO) mice showed leakage from a lesion, resulting from an impaired structure with the loss of the junction complex. Consistently, focal brain injury in the AKAP12 KO mice led to extended inflammation and more severe tissue damage compared to the wild type (WT) mice. Accordingly, our results suggest that AKAP12-positive cells in the fibrotic scar may restrict excessive inflammation, demonstrating certain mechanisms that could underlie the beneficial actions of the fibrotic scar in the new tissue formation stage during the CNS repair process.

## Introduction

In most organ systems, the response to injury can be commonly classified into three distinct stages: inflammation, new tissue formation, and remodeling [1]. The CNS repair process also shows a well-organized cascade of three distinct stages [2]. Inflammation occurs immediately after CNS injuries. Numerous blood-born immune cells infiltrate into the lesion and resident microglia cells are also activated [3]. Such an innate immune system prevents additional infections and regulates the phagocytosis of damaged tissue [4]. In next stage, new tissue formation occurs from days to weeks after injury. Activated proliferating cells derived from various origins migrate to the lesion site and produce chondroitin sulfate proteoglycans (CSPGs) and extracellular matrixes (ECMs), resulting in the formation of the CNS scar which consists of two distinct layers, the fibrotic scar and the glial scar. The fibrotic scar directly surrounds the lesion site and the glial scar forms the boundary between the fibrotic scar and the normal parenchymal tissues [5], [6]. Lastly, the remodeling stage starts about 3 weeks after injury and can be maintained for several months depending on injury. During this stage, newly formed tissues are stabilized and axonal circuits are reconstructed by axonal regeneration [7].

Because the fibrotic scar is the main resource of CSPGs and ECMs which inhibit axonal regeneration, it has been recognized as an obstacle for axonal regeneration during the remodeling stage. Therefore, previous studies on the fibrotic scar mainly focused on blocking the harmful function of the remodeling stage with its inhibition of axonal regeneration [8], [9]. However, the role of the fibrotic scar in the new tissue formation stage remains largely undefined. Recently, it has been identified by several studies that the fibrotic scar is a complex structure composed of various cells which have different properties and origins like meningeal cells and pericytes [5], [10], implying that the fibrotic scar could be multi-functional. Therefore, the roles of fibrotic scar need to be investigated in the new tissue formation stage of the CNS repair process.

AKAP12 is known as a tumor suppressor protein reduced in the metastatic progression of human prostate cancer [11], and loss of the AKAP12 gene induced prostatic hyperplasia in mice [12]. Furthermore, an *in vitro* study showed that the suppression of AKAP12 increases cell motility and invasion [13]. In zebrafish development, AKAP12 regulates the movement of mesodermal cells. AKAP12 morphant embryos exhibited severe extension defects, resulting from the unregulated protrusive activity of paraxial mesodermal cells [14]. Because AKAP12 is crucial for cell motility and stability, which are closely related to scar formation and it is the multifunctional scaffolding protein which serves as a platform for various signals, it is an attractive candidate molecule that integrates scar formation as a result of complex events such as the immune response, migration of various cells, and tissue remodeling.

Here, we show that AKAP12-positive cells participate in formation of the fibrotic scar and that the cells mediate the beneficial role of the fibrotic scar as a barrier through the structure which physically segregates immune cells during the new tissue formation stage of CNS repair. Together with the data of previous studies [8], our findings suggest that the fibrotic scar could have different functions depending on the stage of repair following CNS injury, providing an extended and more nuanced view of the fibrotic scar in CNS injuries.

## Materials and Methods

### 1. Animals

C57BL/6 (Orient Bio Inc., Seongnam, Korea) were used for observations at the serial time points. Breeding colonies of WT and AKAP12 KO mice (C57BL/6 background) [12] were established and used for comparison experiments. All mice were maintained in a SPF room in the animal-housing facilities at the Seoul National University. Animal experiments were approved by the Committee for Care and Use of Laboratory Animals at the Seoul National University, according to the Guide for Animal Experiments edited by the Korean Academy for Medical Sciences.

### 2. Photothrombosis & MCAO

WT and AKAP12 KO mice (C57BL/6 background, male, weighing 23–25 g) were subjected to photothrombosis [15]. Under anesthesia by intraperitoneal injection of Zoletil (30 mg/kg) and Rompun (10 mg/kg), Rose Bengal (Sigma, 0.1 ml/25 g body weight of 10% solution) was injected via the tail vein, which was allowed to circulate for 5 min. After an incision in the scalp was made, the skull was exposed to a cold light source (Zeiss FL1500 LCD, 150W, 1 mm diameter) for 20 min. The position of the optic center was 2.5 mm to the left and 2.5 mm to the back of the bregma. The scalp was sutured and the mice were allowed to regain consciousness.

Male Wistar rats, weighing 280–310 g, were subjected to MCAO/R using the modified method of the intraluminal nylon suture technique [16], [17]. Briefly, under a surgical microscope and general anesthesia, the left common carotid artery (CCA) was exposed via a ventral midline incision. The CCA, external carotid artery (ECA) and pterygopalatine artery were ligated with 5-0 black silk. A 4-0 monofilament nylon (Doccol Corp.) was advanced approximately 23 mm from the ICA origin into the MCA. Reperfusion was achieved by pulling on the suture until resistance was felt.

### 3. Immunofluorescence

Anesthetized mice were perfused with 0.1 M PBS (pH 7.4). Isolated brains were fixed with 4% PFA at 4°C and dehydrated with serial gradients of sucrose. Cryostat sections of 10 µm were attached on the gelation coated slide and 30 µm sections were stored in the preservation solution for floating staining. The samples were stained with primary antibodies against AKAP12 (I. Gelman, Roswell Park Cancer Institute), E-cadherin, CD31, CD45 (BD), GFAP, fibronectin, MPO (DAKO), GS-lectin, Occludin, ZO-1 (Invitrogen) and IBA1 (WAKO) overnight at 4°C followed by Alexa 488, 546 and 350 (Invitrogen) secondary antibodies at RT for 1 h. Nuclear-staining was performed with Hoechst 33342 (Molecular Probes). Images were obtained with an ApoTome microscope (Carl Zeiss, Axiovert M200) or confocal microscopy (Carl Zeiss, LSM700).

### 4. Evans blue permeability assay

Four mice per WT and KO at each time point were used for comparison. Under anesthesia with 4% isoflurane, Evans blue solution (Sigma, 2% in saline; 4 mL/kg) was injected via the tail vein. After circulation for 2 h, brains were extracted and dissected into hemispheres. Each hemisphere was homogenized (Tissue Tearor, 50 s; speed 35) in 1 mL (total volume) of 0.1 M PBS. The supernatant was collected and mixed with trichloroacetic acid solution (Sigma, 50% in saline). After centrifugal precipitation, the supernatant was extracted and the absorbance was measured at 610 nm with a spectrophotometer.

### 5. Luxol Fast Blue (LFB) staining & Triphenyltetrazolium chloride (TTC) staining

Four mice per WT and KO group were used for comparison. Six representative sections were selected at the lesion site 540 µm in diameter and stained with LFB solution (Sigma). Serial brain slices (1 mm thickness) near the lesion site were incubated for 30 min at 37°C in 0.05% TTC (Sigma) solution. After washing in PBS, stained slices were fixed in 4% PFA for 30 min. The ratio of the LFB (−) region and TTC (−) region to the total brain was analyzed with image analysis software (NIH-Image J).

### 6. Western blotting

The lesion tissues from four mice per group were homogenized and lysed in buffer [20 mM Tris-HCl (pH 7.5), 150 mM NaCl, 1 mM Na_2_EDTA, 1 mM EGTA, 1% Triton, 2.5 mM sodium pyrophosphate, 1 mM beta-glycerophosphate, 1 mM Na_3_VO_4_, 1 µg/ml leupeptin, and protease inhibitor cocktail]. Western blot analysis was performed as described previously [18]. Immunoblotting was performed using primary antibodies against AKAP12 (Santa Cruz, I. Gelman, Roswell Park Cancer Institute), α-tubulin (InnoGenex), E-cadherin (BD), ZO-1 and Occludin (Invitrogen).

### 7. Genomic PCR

Genomic PCR analysis was performed as described previously [12]. Sequences for the primers used are as follows. WT allele (F) 5′-CGC TGT ACT ACT AAG GAG AGT GTT ACG-3′ and (R) 5′-CCT CCT GGG TCT CAG CCA GTT TCT CAG GGG-3′, KO allele: (F) 5′-CGG CTG GGT GTG GCG GAC CGC TAT CAG GAC ATA GCG-3′ and (R) 5′-CTC AGC CTT TGC CAG AAT AGG CAC TGC CCC-3′

### 8. Data analysis and statistics

Quantification of the band intensities was analyzed with Image J and normalized to the density of GAPDH and α-tubulin. Statistical analysis was done with an unpaired two-tailed Student t-test for single comparisons and ANOVA followed by post hoc analysis with Tukey-Kramer test for multiple comparisons. P<0.05 was considered statistically significant.

## Results

### AKAP12 is highly expressed in the fibrotic scar surrounding the lesion during CNS repair process

**Figure 1:**
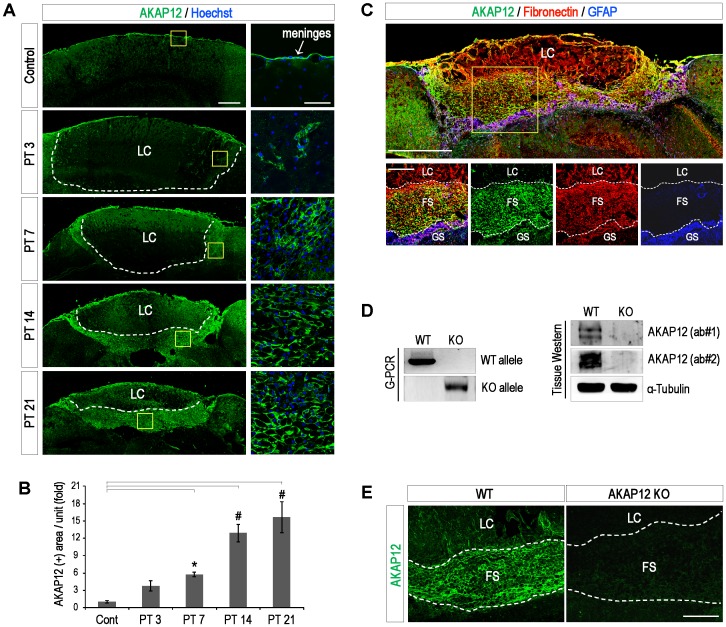
AKAP12 is highly expressed in the fibrotic scar surrounding the lesion in the process of CNS repair. (A) Brain sections were stained with antibody against AKAP12. AKAP12 was highly expressed in the normal meninges, and the number of AKAP12-positive cells in the scar tissue increased over time. Yellow boxes were magnified in right panels. Scale bar: 500 µm (large panels), 50 µm (magnified panels) (B) The AKAP12-positive area of three representative sections per mouse was analyzed using Image J. (Mean ± S.D.; n = 4 mice per each time; P*<0.05, P#<0.001) (C) Mouse brains were harvested at 21 days after photothrombotic injury. Brain sections were stained with antibody against AKAP12, GFAP (a marker for the glial scar) and fibronectin (a marker for the fibrotic scar). Yellow box was magnified in lower panels. Scale bar: 500 µm (largest panel), 200 µm (magnified panels) (D) AKAP12 gene KO was confirmed through genomic PCR and tissue western blotting. PCR analysis of mouse brain DNA showing the AKAP12 WT and KO alleles in the WT or AKAP12 KO mice and immunoblotting analysis of the brain lysates from the WT and KO mice using two different AKAP12 antibodies. (E) Mouse brains were harvested at 21 days after photothrombotic injury. The specificity of the AKAP12 signal was supported by lack of AKAP12 staining in the fibrotic scar in the AKAP12 KO mice. Scale bar: 200 um [LC: lesion core, FS: fibrotic scar, GS: glial scar].

To identify the role of AKAP12 in the repair process after CNS injury, we sampled mice brains at serial time points after focal brain injury induced by photothrombosis, and examined the AKAP12 level during CNS repair. Immumnostaining data shows that AKAP12 was highly expressed in the normal meninges (Fig. 1A_Control) and the number of AKAP12-positive cells increased around the lesion tissue remarkably over time (Fig. 1A and B). Finally, AKAP12-positive cells formed the distinct layer bound to the lesion core (Fig. 1A_PT 14 and PT21).

Similar to previous studies [19], [20], we observed that photothrombotic injury also induced the formation of the CNS scar which consists of two distinct layers, the fibrotic scar (fibronectin-positive) and the glial scar (GFAP-positive) surrounding the lesion site (Fig. 1C largest panel) and that AKAP12-positive cells were primarily located in the fibrotic scar tissue (Fig. 1C magnified panels). For the *in vivo* validation of the role of AKAP12-positive cells in the fibrotic scar, we introduced the AKAP12 KO mice system [12]. Prior to the *in vivo* validation, the knock-out of the AKAP12 gene was confirmed by comparing the brain tissues of WT and AKAP12 KO mice using genomic PCR and western blotting (Fig. 1D). As expected, the AKAP12 signals specifically disappeared in the fibrotic scar of the AKAP12 KO mice (Fig. 1E). These data show that AKAP12-positive cells are found in the fibrotic scar.

### AKAP12 KO mice showed leakage from the lesion with abnormal morphology of the fibrotic scar

**Figure 2:**
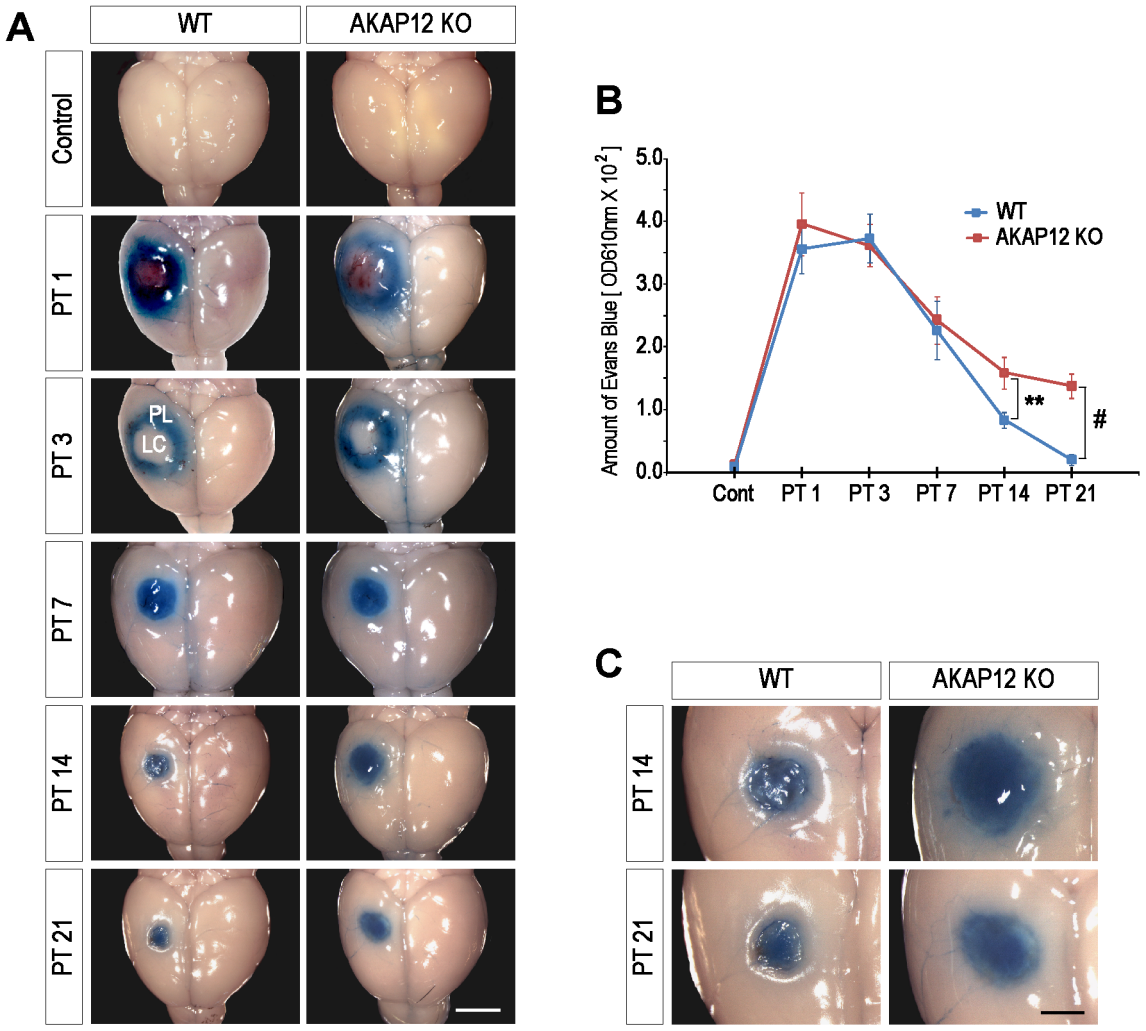
AKAP12 KO mice showed leakage from the lesion with abnormal morphology of the fibrotic scar. (A) Evans blue extravasation assay. Significant differences between the WT and AKAP12 KO mice were observed from day 14 after injury. Scale bar: 2 mm (LC: lesion core, PL: peri-lesion) (B) Evans blue was extracted from the brain tissue, and the amount of Evans blue was quantitatively analyzed by measuring the OD (mean ± S.D.; n = 4 mice per WT and KO at each time point; P**<0.01, P#<0.001). (C) The lesion site of the WT mouse was tightly separated with the normal tissue on day 14 and 21 after injury without any leakage. However, the AKAP12 KO mice showed an unclear boundary between the lesion site and normal tissue with a dispersion of Evans blue. Scale bar: 1 mm.

Photothrombosis induces thrombus in most blood vessels of the illuminated lesion core (LC) [15], [21] and it completely blocks the circulation through the blood vessel (Supplementary Fig. 1A). Thus, immediately after injury, immune cells only infiltrate through the peri-lesion (PL) site not the LC site, resulting in blood-brain barrier (BBB) breakage by inflammation that primarily occurs at the PL site. Therefore, Evans blue extravasation in the inflammation stage (PT1∼PT3) was observed in the shape of a ring with the lesion core as the center (Fig. 2A-WT group_ PT1 and PT3). This pattern of Evans blue extravasation disappeared before reaching day 14 after injury (Fig. 2A-WT group_ PT7, PT14 and PT21), implying that spontaneous recovery of impaired BBB was primarily occurred during this period [22], [23].

During the new tissue formation stage (PT14∼PT21), infiltrating immune cells cause the lesion tissue to be destroyed by phagocytosis, and the newly formed scar tissue separates the lesion from normal tissue. Hoechst staining and differential interference contrast (DIC) images in Supplementary Fig. 1B show that the cell number and the density of lesion tissue are very low compared to normal brain tissues at 21 days after injury, while many cells form scar tissue with high density around the LC site. Therefore, Evans blue dye in the cerebrospinal fluid (CSF) accumulated in the loose lesion tissue without dispersing across the fibrotic scar (Fig. 2A-WT_PT14, PT21, and Fig. S1C). Additionally, vessels were rarely observed in the LC, showing that the accumulated dye in LC did not result from BBB leakage generated by the newly formed vessels (Fig. S1D). These results suggest that the fibrotic scar could function as a barrier in the new tissue formation stage of repair (PT14∼PT21).

Likewise, BBB breakage (Evans blue extravasation shaped of ring) seemed to have mainly recovered before reaching day 14 after injury in our injury model, and BBB breakage was not observed near the lesion site on day 14 after injury, the time when the fibrotic scar may begin to function as a barrier (Fig. 2A-WT group). Therefore, although the Evans blue permeability assay has been used for the quantification of BBB breakage, in this experiment, we can evaluate the barrier function of the fibrotic scar from day 14 after injury using the assay.

As a result, there was no difference from day 1 to 7 after injury between the WT and AKAP12 KO mice; however, significant differences emerged from day 14 after injury (Fig. 2A). These results correlate with the previous data (Fig. 1A and B) that the AKAP12 level significantly increased from day 14 after injury. Extravasation of Evans blue into the brain tissue of the AKAP12 KO mice was quantitatively increased 1.9 fold on day 14 and 6.9 fold on day 21 compared to the WT mice (Fig. 2B). While the lesion site of the WT mice was tightly separated with normal tissue by a solid structure on day 14 and 21 after injury, the AKAP12 KO mice showed an unclear boundary between the lesion site and the normal tissue with a dispersion of Evans blue near the lesion site (Fig. 2C). These data suggest that AKAP12 KO mice may have an impaired fibrotic scar that allows the dispersion of Evans blue dye.

### AKAP12-positive cells form a structure that restricts immune cells in the fibrotic scar

Interestingly, the structure formed by AKAP12-positve cells was not just a simple layer made by cell adhesion. Through histological analysis at serial time points after photothrombotic injury, we found that AKAP12-positve cells form the structure trapping immune cells in the fibrotic scar.

**Figure 3:**
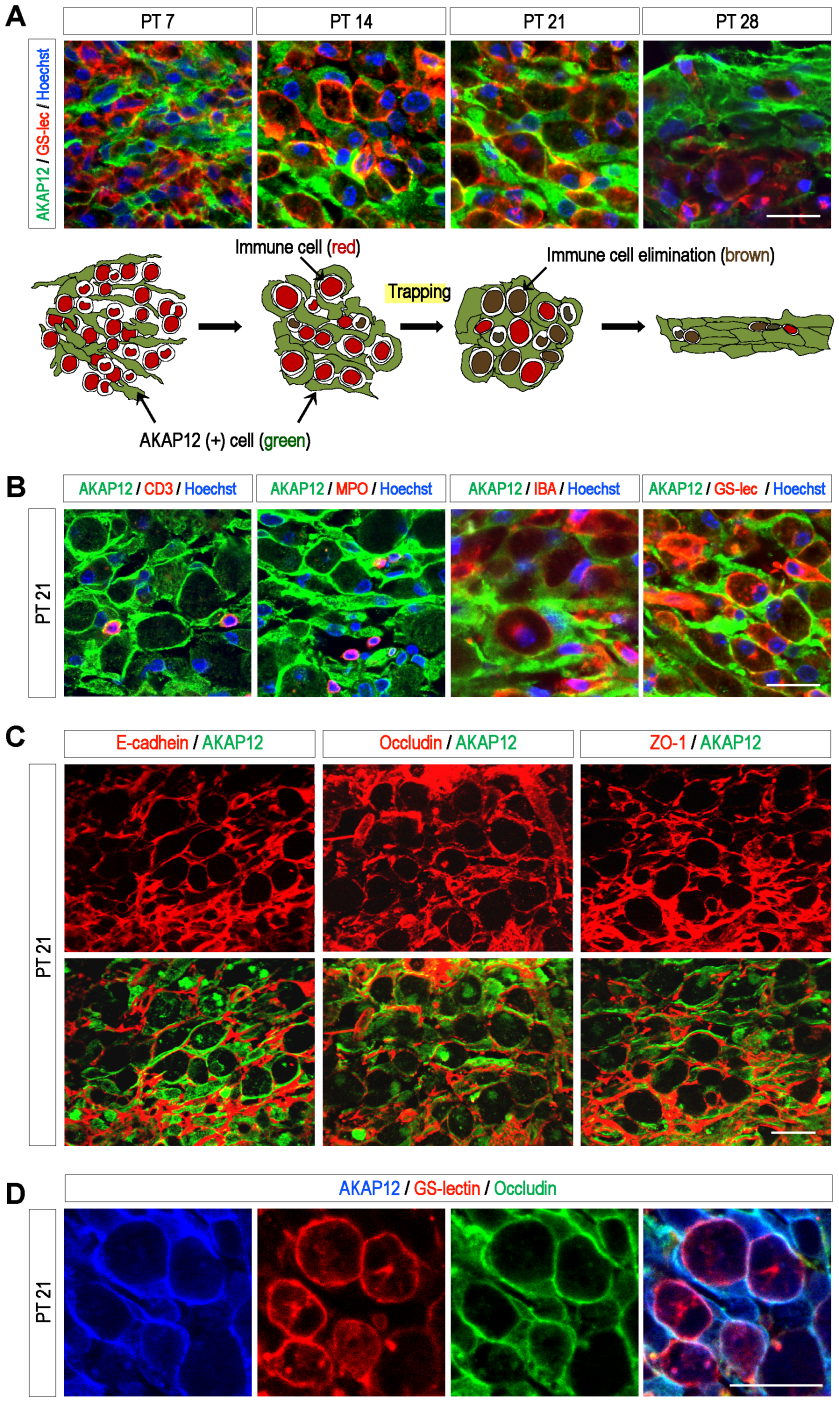
AKAP12-positive cells form a structure to restrict immune cells in the fibrotic scar. (A) Mouse brains were harvested at serial time points after photothrombotic injury. Brain sections were stained with antibodies for AKAP12 and GS-lectin (a marker for monocytes and macrophages). Nuclei were stained with Hoechst. Scale bar: 20 µm (B) The structure formed by AKAP12-positive cells traps most types of immune cells. Brain sections were stained with antibodies for AKAP12, CD3 (a marker for T cells), MPO (a maker for neutrophils), and IBA1 (a marker for monocytes and macrophages) and GS-lectin (a marker for monocytes, macrophages). Scale bar: 20 µm (C) Mouse brains were harvested at 21 days after photothrombotic injury. AKAP12-positive cells in the fibrotic scar expressed Occludin, E-cadherin and ZO-1 which permit their function as a physical barrier. Scale bar: 40 um. (D) Brain sections were triple-stained with antibodies for AKAP12, GS-lectin and Occludin. Scale bar: 20 µm.

On day 7 after injury, pseudomorphous AKAP12-positive cells were embedded among numerous immune cells infiltrated into the lesion site, and the cells linked to each other around the immune cells over time. On day 14 after injury, the AKAP12-positive cells formed an interesting structure, shaped like a sponge, trapping immune cells. Thereafter, the cells attached to each other eliminating the immune cells and finally formed a thick layer (Fig. 3A). A lot of phagocytic monocytes and macrophages along with a few T cells and neutrophils were observed in the fibrotic scar on day 21 after injury, and most types of immune cells were trapped in the structure formed by the AKAP12-positive cells (Fig. 3B). To confirm that the immune cell- trapping structure by AKAP12-positive cells is also formed in another injury model, we utilized the rat middle cerebral artery occlusion (MCAO) model and found the formation of a similar structure on day 21 after injury. The AKAP12-positive cells formed an immune cells-trapping structure in the lesion near the meninges (Fig. S2_a, b). However, this structure was not observed deep inside the MCAO lesion (data not shown). It is thought that there are some differences for more widespread infarct size in MCAO compared to PT. These results suggest that our findings are not an artifact of the photothrombosis model, and that AKAP12-positive cells could be involved in a meningeal response to injury.

Tight junction and adhesion proteins like Occludin, ZO-1 and E-cadherin are known to be essential for the maintenance of various biological structures by connecting between associated cells, and allow for the function of a physical barrier by blocking the flow of substances [24], [25], [26]. Interestingly, the AKAP12-positive cells, which form the structure trapping the immune cells, strongly expressed such tight junction and adhesion proteins (Fig. 3C). Collectively, triple staining data in Fig. 3D show that AKAP12-positive cells formed structures by connecting to each other through the tight junction protein Occludin, following which, GS lectin-positive immune cells became embedded in the structure. For these properties of the AKAP12-positive cells such as tightly enclosing the immune cells and highly expressing tight junction proteins, the structure formed by the AKAP12-positive cells is expected to function as a barrier to restrict inflammation in the fibrotic scar.

### AKAP12 KO mice showed an impaired fibrotic scar structure with loss of junction complexes

**Figure 4:**
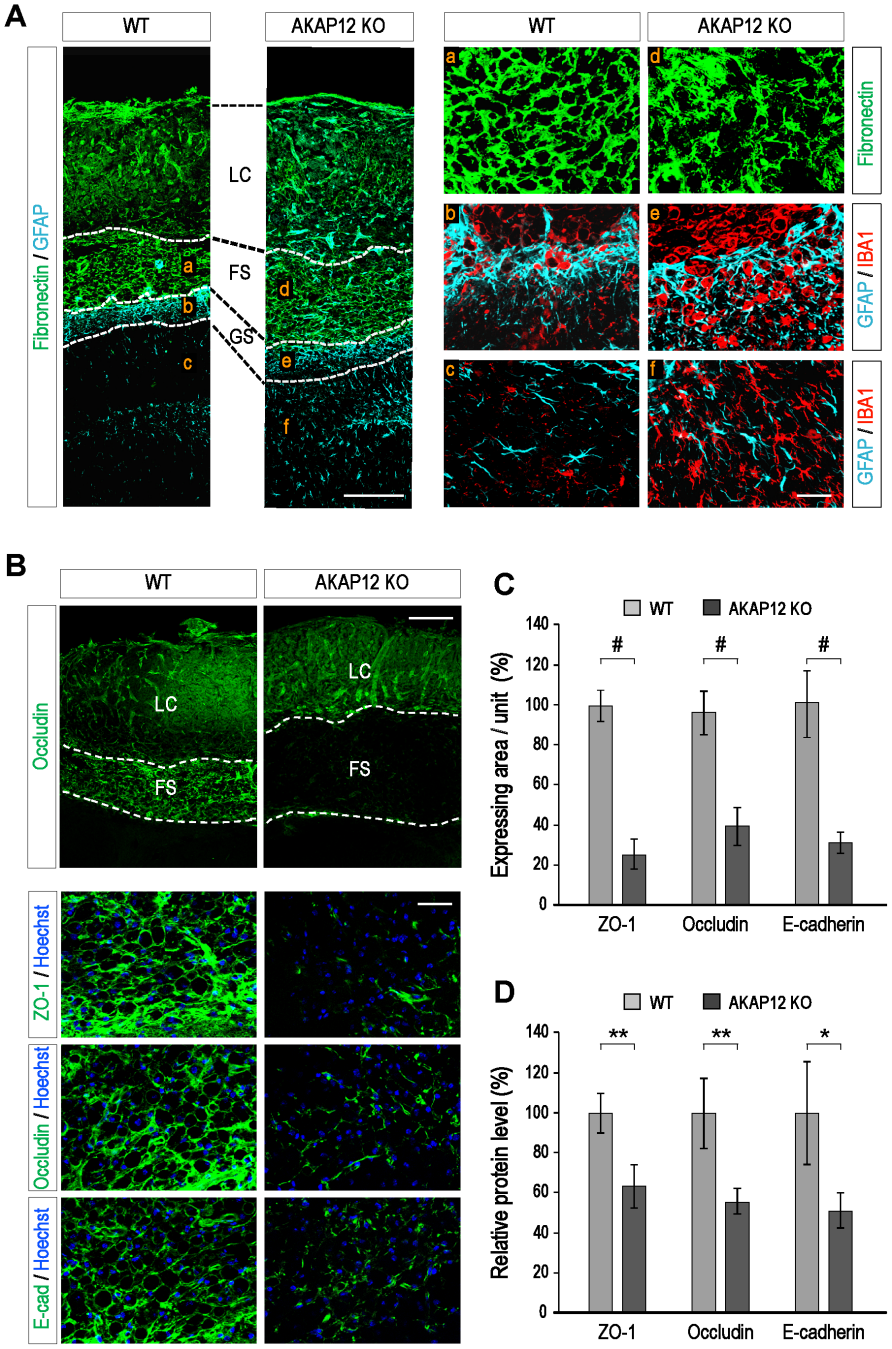
AKAP12 KO mice showed an impaired fibrotic scar structure with loss of junction complexes. (A) The structure of the scar tissue was unstable in the AKAP12 KO mice (a/d). More IBA1-positive immune cells infiltrated across the fibrotic scar in the AKAP12 KO mice (b/e). AKAP12 KO mice showed much more hypertrophic GFAP-positive astrocytes and IBA1-positive microglial cells near the fibrotic scar (c/f). Scale bar: 200 µm (left panel), 40 µm (right panel) (B) The expression of junction proteins in the fibrotic scar was reduced in the AKAP12 KO mice compared to the WT mice on day 21 after injury. Brain sections were stained with antibodies against ZO-1, Occludin, and E-cadherin. The lower three columns are magnified images of the fibrotic scar. Scale bar: 200 µm (upper panel), 40 µm (lower panel) (C) The experimental condition was same for panel b. For each marker, the immunoflouresence intensity of three representative slides per mouse were analyzed using Image J. (Mean ± S.D.; n = 4 mice per each time; P#<0.001) (D) The global level of junction proteins were decreased in the AKAP12 KO mice. The lesion tissues were harvested from the brains of mice on day 21 after injury and the tissue lysates were analyzed by Western blot (Mean ± S.D.; n = 4 mice per WT and KO; P*<0.05, P**<0.01). [LC: lesion core, FS: fibrotic scar, GS: glial scar].

To validate the function of the AKAP12-positive cells in the fibrotic scar *in vivo*, histological differences in the fibrotic scar were compared between WT and AKAP12 KO mice after photothrombotic brain injury. The fibronectin staining data reveals that the scar structure of the AKAP12 KO mice was loosely assembled on day 21 after injury (Fig. 4A a/d). Consequently, more IBA1-positive immune cells infiltrated into the parenchymal tissues across the fibrotic scar (Fig. 4A b/e). Furthermore, IBA1-positive resident microglia and GFAP-positive astrocytes near the fibrotic scar in the AKAP12 KO mice showed a hypertrophic morphology (Fig. 4A c/f), implying that inflammatory materials were discharged from the fibrotic scar. Immunostaining data shows that the expressions of ZO-1, Occludin and E-cadherin were significantly reduced in the fibrotic scar of the AKAP12 KO mice compared to the WT mice (Fig. 4B and C).When we analyzed lysates from the lesion tissues harvested as described in Fig. S3, the results of western blot revealed reduced levels of junction proteins in the lesion tissue of the AKAP12 KO mice similar to the result of immunostaining data, (Fig. 4D). These data suggest that AKAP12 is crucial for maintaining the junction complex of the cells which form scar barrier; and such breakage of the junction complex explains well the impaired scar structure (Fig. 4A a/d) and the scar barrier not functioning in the AKAP12 KO mice (Fig. 2C).

### AKAP12 KO mice showed extended immune cell infiltration and tissue damage compared to the WT

**Figure 5:**
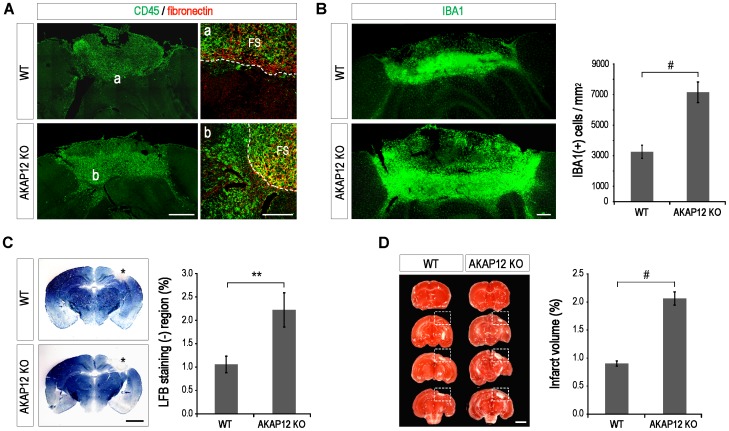
AKAP12 KO mice showed larger demyelinated and infarcted tissue compared to WT from the extended infiltration of immune cells. (A) The areas infiltrated by CD45-positive leukocytes increased in the AKAP12 KO mice on day 21 after injury. Brain sections were co-stained with antibodies against fibronectin and CD45 (the marker for pan leukocytes). Scale bar: 500 µm (left panel), 200 µm (magnified panel) (FS: fibrotic scar) (B) The AKAP12 KO mice showed more severe inflammation compared to the WT mice. The areas infiltrated by the IBA1-positive immune cells and the number of IBA1-positive cells per unit area near the lesion site increased in the AKAP12 KO mice on day 21 after injury (mean ± S.D.; n = 6 mice per WT and KO and 3 positions per mouse; P#<0.001). Scale bar: 200 µm (C) The AKAP12 KO mice showed increased demyelination compared to the WT. Luxol fast blue (LFB) staining was performed to evaluate demyelination (the asterisk region) in the injured brain (mean ± S.D.; n = 4 mice per WT and KO; P**<0.01). Scale bar: 2 mm (D) The AKAP12 KO mice showed increased tissue damage compared to the WT. Triphenyltetrazolium chloride (TTC) staining to evaluate the infarct volume (Mean ± S.D.; n = 4 mice per WT and KO; P#<0.001). Scale bar: 2 mm.

To confirm whether AKAP12-regulated pathways are beneficial during the recovery phase after brain injury, the extent of immune cell infiltration and tissue damage were compared between the WT and AKAP12 KO mice. As shown in Fig. 5A, while most CD45-positive immune cells were restricted within the fibrotic scar in the WT mice, many more immune cells spread further into the neural parenchyma in the AKAP12 KO mice across the fibrotic scar. Moreover, many more cells, which express IBA1, a marker for macrophage and microglia, were observed near the lesion site of the AKAP12 KO mice compared to the WT mice (Fig. 5B).

To compare the degree of inflammation near the scar, we counted only spherical cells excluding inactive microglia which have a ramified form among the IBA1-positive cells. The total number per unit (1 mm^2^) of IBA1-positive cells near the lesion site increased 2.2 fold in the AKAP12 KO mice compared to the WT mice on day 21 after injury [WT: 3265±424, AKAP12 KO mice: 7153±677] (Fig. 5B graph), implying that inflammation is more severe in the AKAP12 KO mice. Interestingly, the immune cell population, which has a small size, was much higher in the AKAP12 KO mice relative to the WT mice, implying the possibility that new immune cells continue to infiltrate across the scar barrier. Because severe inflammation could lead to extended tissue damage in the CNS, we compared tissue damage between the WT and AKAP12 KO mice on day 21 after injury by measuring demyelination and infarct size. The extent of demyelination was evaluated with Luxol fast blue (LFB) staining. The proportion of LFB-negative regions to the total brain increased 2.1 fold in the AKAP12 KO mice [WT: 1.05±0.18%, AKAP12 KO: 2.22±0.37%] (Fig. 5C). Finally, the extent of infarct size was evaluated with triphenyltetrazolium chloride (TTC) staining. The ratio of TTC-negative regions to the total brain increased 2.3 fold in the AKAP12 KO mice compared to the WT mice on day 21 after injury [WT: 0.90±0.05%, AKAP12 KO: 2.06±0.12%] (Fig. 5D). These results strongly suggest that AKAP12 is important in the formation and maintenance of the scar barrier, which acts as a potentially protective physical barrier during recovery after brain injury.

## Discussion

There have been experimental limitations in verifying the *in vivo* function of the fibrotic scar using conditional KO mice due to the absence of information about the exact cell types comprising the fibrotic scar and their marker proteins. Therefore, previous studies on CNS scars primarily concentrated on the glial scar composed of astrocytes which has specific marker proteins like GFAP [27], [28]. However, through recent studies, meningeal cells [5] and pericytes [10] were identified as cell types found in the fibrotic scar, and it has been suggested that the fibrotic scar also has important roles in CNS repair, and it could be a potent target for therapeutics [9], [29]. Therefore, it is expected that the fibrotic scar will become a subject of great interest in the field of CNS repair.

Although the function of the fibrotic scar was reported to be harmful as an obstacle for axonal regeneration in the remodeling stage of repair [8], all three stages of repair, inflammation, new tissue formation, and tissue remodeling, have shown both detrimental and beneficial effects [6], [30]. Furthermore, the fibrotic scar is a complex structure comprised of multiple cell types which have different properties [5], [10]; therefore, it could be multi-functional depending on the stage of CNS repair. From this viewpoint, our findings suggest the possibility that the fibrotic scar has beneficial roles in the new tissue formation stage of repair, which are mediated by AKAP12-positive cells.

Based on the serial observations in our injury model, the proportion of AKAP12-positive cells in the fibrotic scar increased over time, and they finally formed the structure trapping the immune cells infiltrated into the lesion site by linking to each other. Interestingly, the AKAP12-positive cells highly expressed junction and adhesion proteins like Occludin, ZO-1, and E-cadherin. These proteins are known to be essential for the barrier function of biological structures including the BBB and the epithelium by making junction complexes which provide strong tightness between cells. Therefore, it is expected that the AKAP12-positive cells did not simply encircle immune cells but restrict them from the normal parenchymal tissue. Our *in vivo* validation data strongly support this possibility by showing extended inflammation and tissue damage with the loss of the junction complex in the fibrotic scar of the AKAP12 KO mice. These new findings show the mechanisms that underlie the beneficial actions of the fibrotic scar in the new tissue formation stage of the CNS repair process.

Additionally, Occludin, ZO-1, and E-cadherin, expressed by AKAP12-positive cells are also known as the most well-known makers for epithelial cells and the AKAP12 KO mice showed a reduced level of these junction proteins in the fibrotic scar. Furthermore, we observed that the AKAP12 knock-down decreased the junction proteins in several epithelial cell lines (data not shown). These data imply that the origin of the AKAP12-positive cells could be cells with epithelial properties and AKAP12 is expected to be crucial for maintaining epithelial properties by regulating the expression of junction proteins.

**Figure 6:**
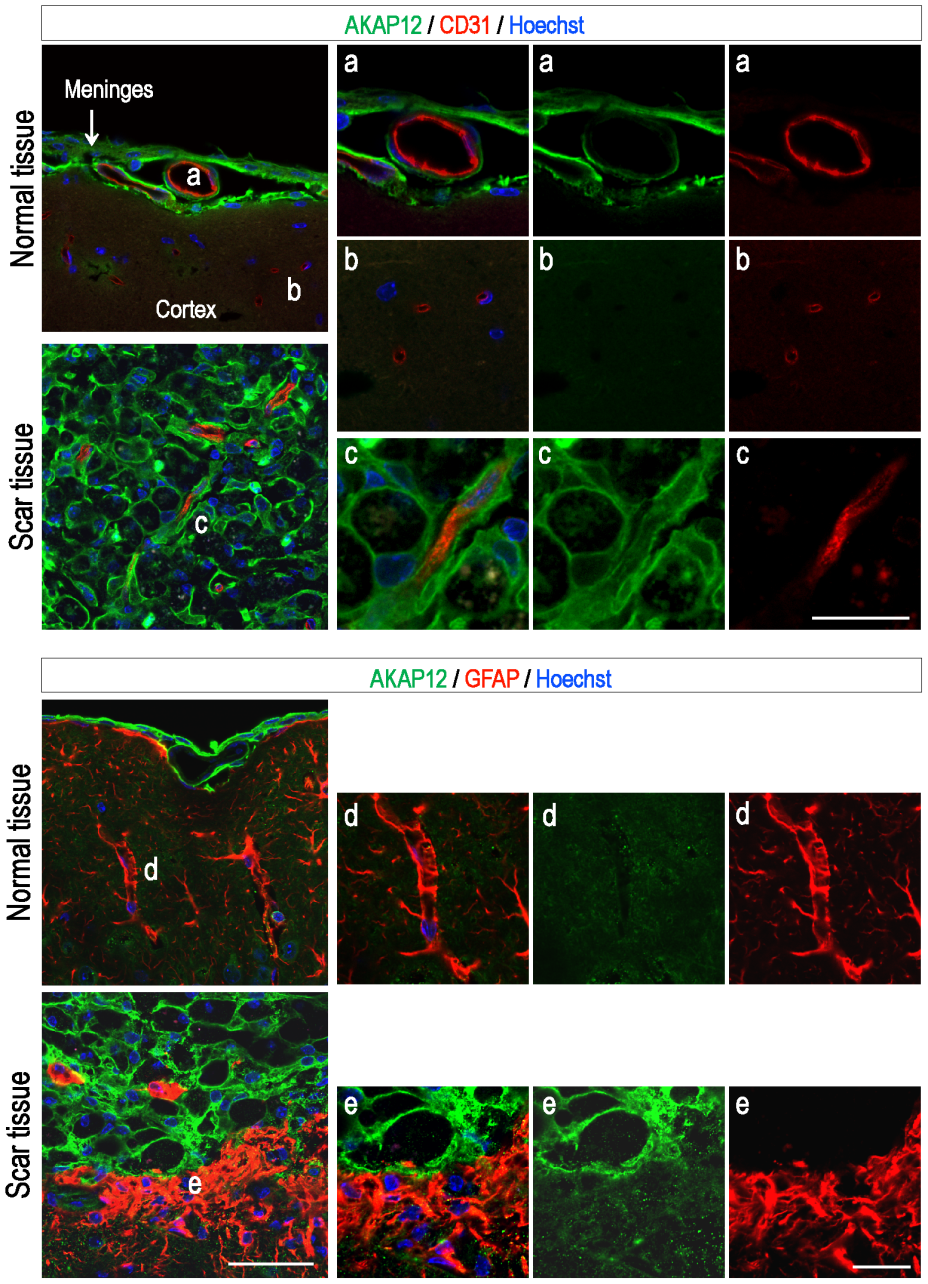
The expression of AKAP12 is very low in the endothelial cells and astrocytes of the adult mouse brain. The mouse brain was extracted on day 21 after injury, and brain sections were stained with antibodies for AKAP12, GFAP (the marker for astrocyte) and CD31 (the marker for endothelial cell). Nuclei were counterstained with Hoechst 33342. AKAP12 was little expressed in the endothelial cells of vessels in normal meninges (panel a) and cortex (panel b). Endothelial cells of vessels in the fibrotic scar also hardly expressed AKAP12 (panel c). AKAP12 level was very low in normal astrocytes wrapping vessels (panel d). The expression of AKAP12 seems to be slightly increased in reactive astrocytes forming the glial scar. However, it was insignificant compared to that of the meningeal cells (panel e). Scale bar: 50 µm (leftmost panels), 20 µm (panel a, b, d and e have same magnification except panel c).

Meanwhile, inflammation, gliosis, scar formation after brain injury and neurodegeneration are complex multiphasic processes [30], and we used the general KO system for *in vivo* validation of AKAP12. Therefore, it must be acknowledged that various cells such as endothelial cells, meningeal cells, astrocytes, microglia, and pericytes may also play some roles in the effects observed in the AKAP12 KO. Particularly, we have reported that AKAP12 regulates vessel integrity and BBB formation during the development of zebrafish and mouse, respectively [31], [32]. However, there seems to be differences in the function of AKAP12 between development and adult pathophysiological conditions. In contrast to the zebrafish embryo, the expression of AKAP12 was very low in the endothelial cells (ECs) of adult mouse brain vessels (Fig. 6 panel a and b), and scarcely changed in the ECs until day 21 after injury (Fig. 6 panel c) Normal astrocytes wrapping vessels in the BBB structure also hardly expressed AKAP12 in the normal adult mouse brain (Fig. 6 panel d). Although the expression of AKAP12 was slightly increased in activated astrocytes to form the glial scar near the lesion site, it was insignificant compared to that of the cells to form the fibrotic scar (Fig. 6 panel e). In our model, the leakage of microvessels primarily peaked on day 3 after injury, and gradually recovered until day 7 after injury (Fig. 2A: Evans blue extravasation assay). If AKA12 functions in vessel integrity and the BBB, there have to be differences in the microvessel leakage between WT and AKAP12 KO mice during this period. However, differences were not observed in this period but from day 14 after injury, the time when the fibrotic scar may begin to function as a barrier. Moreover, it is expected that differences in vessel integrity and BBB stability could affect the penumbra. However, the size of the penumbra was also not changed by AKAP12 KO (Fig. S4), consistent with the Evans blue extravasation assay. These data suggest that the effect of the AKAP12 KO may primarily result from a malfunction in the protective fibrotic scar rather than from the loss of vessel integrity and BBB instability under adult pathophysiological condition. However, since it cannot be completely excluded that AKAP12 KO could affect vessel integrity and BBB stability, further studies using an endothelial cell specific AKAP12 KO system are needed to verify the possibility.

The fibrotic scar is expected to be the structure of versatility due to different properties of each cell type forming the fibrotic scar. In this respect, our results show that AKAP12-positive cells in the fibrotic scar have the potential to isolate immune cells, implying that the fibrotic scar could function as a physical barrier to restrict inflammation. Together with previous studies reporting that the fibrotic scar inhibits axonal regeneration during the stage of remodeling, such findings provide an extended view that fibrotic scar could have different functions depending on the stages of CNS repair. Therefore, for targeting the fibrotic scar to promote recovery after brain injury, coordinated approaches are necessary to maintain the protective role of the fibrotic scar in the early stage of repair and to block the destructive pathways that inhibit axonal regeneration in the later stage of repair. Furthermore, follow-up studies are warranted to determine how the protective AKAP12 mechanisms intersect with the destructive pathways such as those involving inhibitory matrix molecules and to explore how these mechanisms may be therapeutically targeted to promote repair and recovery after brain injury.

## Supporting Information

Figure S1 **The histological characteristics of the lesion core tissue.** (A) 2% Evans blue saline solution (100 µL) was injected via the tail vein at 3 days after photothrombotic injury. After circulation for 2 h, the brains were extracted. The unstained lesion core shows that photothrombosis completely blocks the circulation through the blood vessel. Scale bar: 5 mm (B) Mouse brains were harvested at 21 days after photothrombotic injury. Brain sections were stained with Hoechst solution for nuclear staining (pseudo-colored green). Scale bar: 500 µm (upper panel), 200 µm (magnified images) (C) Evans blue saline solution was injected at 21 days after photothrombotic injury. Mice were perfused after circulation for 2 h, and the brains were extracted. The brain section shows that Evans blue dye was restricted within the lesion core without dispersion across the scar tissue. Scale bar: 1 mm (D) Mouse brains were extracted on day 21 after injury, and the brain sections were stained with antibodies for CD31 (the marker for endothelial cell). Vessels were rarely observed in the lesion core where the dye accumulated. Scale bar: 500 µm [N: normal tissue, LC: lesion core, FS: fibrotic scar)].
(TIF)

Figure S2 **AKAP12-positive cells formed immune cell trapping structures in rodent middle cerebral artery occlusion (MCAO) injury models.** The structure formed by AKAP12-positive cells is also observed in rodent middle cerebral artery occlusion (MCAO) injury models. Rat brains were harvested on day 21 after MCAO injury. [a] is the boundary of the lesion site and [b] is the fibrotic scar. Scale bar: 200 µm (left panel), 40 µm (magnified right panel), 40 µm (lower panel).
(TIF)

Figure S3 **The method to define and collect the lesion tissue.** (A) After perfusion, the lesion site was easily distinguishable from the normal tissue. Injured mouse brains were coronal-dissected at the inside from the lesion boundary with a razor blade in a mold. Scale bar: 1 cm, 1 mm (magnified image) (B) Since the scar tissue is white compared to adjacent tissues due to its high cell density, the scar tissue becomes the border line between the normal and the lesion tissue. To harvest both the lesion and scar tissue, brain coronal sections were dissected with a razor blade in three directions along the outside of the scar tissue. Scale bar: 1 mm.
(TIF)

Figure S4 **AKAP12 KO did not affect to the penumbra.** Mouse brains were extracted at 1 day after injury, and serial brain slices (2 mm thickness) near the lesion site were incubated for 30 min at 37°C in 0.05% TTC solution. Pictures of the brain slices were taken after fixation with 4% PFA and analyzed using Image J program. (Mean ± S.D.; n = 4 mice per WT and KO; an unpaired two-tailed Student *t*-test: NS: not significant) Scale bar: 2 mm.
(TIF)
