# Supplementary figures and images for: AKAP12 Mediates Barrier Functions of Fibrotic Scars during CNS Repair
Source: PLoS One. 2014 Apr 23;9(4):e94695. doi: 10.1371/journal.pone.0094695 (PMC3997571; doi:10.1371/journal.pone.0094695)

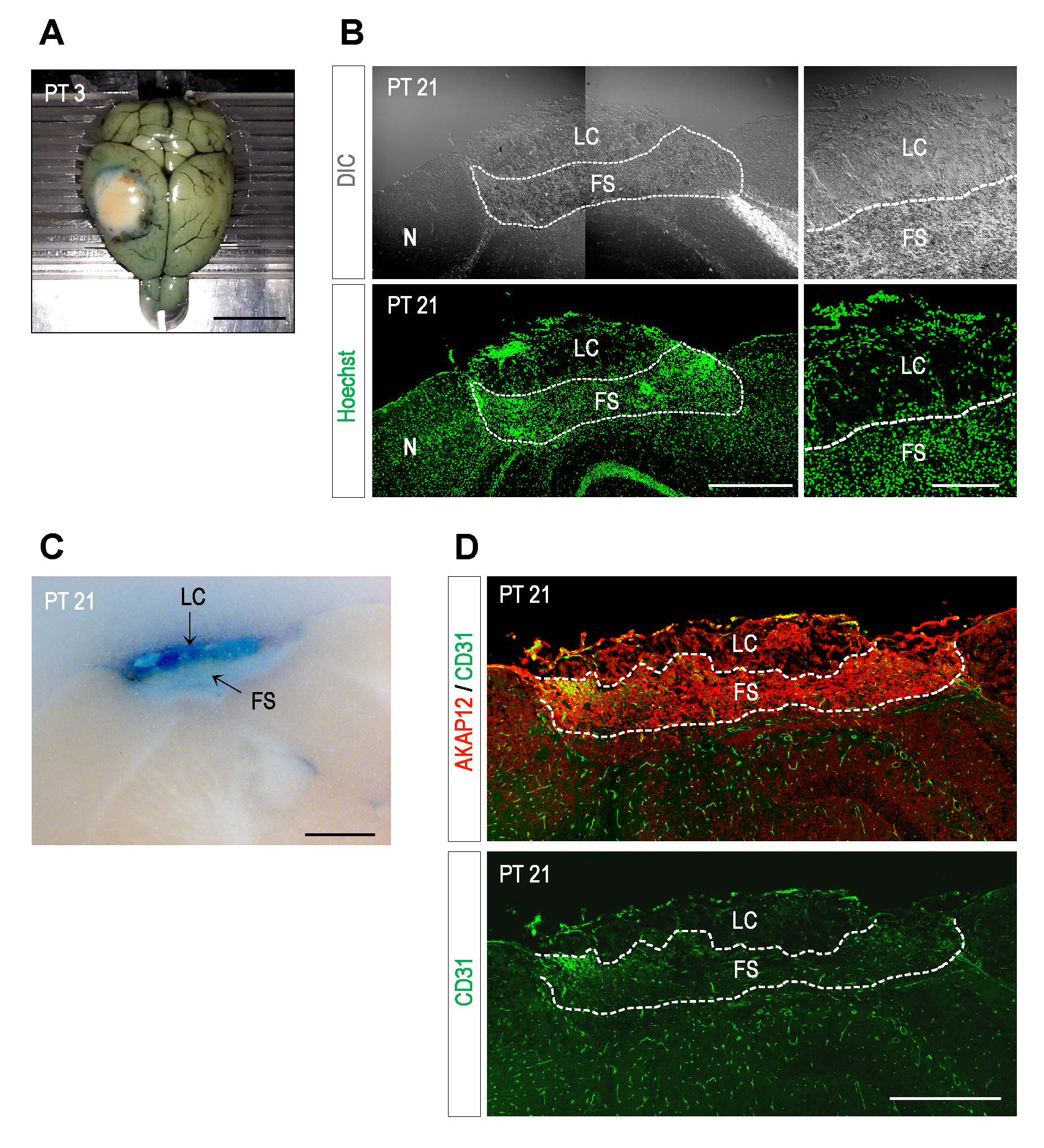

Supplement: Figure S1 — The histological characteristics of the lesion core tissue. (A) 2% Evans blue saline solution (100 µL) was injected via the tail vein at 3 days after photothrombotic injury. After circulation for 2 h, the brains were extracted. The unstained lesion core shows that photothrombosis completely blocks the circulation through the blood vessel. Scale bar: 5 mm (B) Mouse brains were harvested at 21 days after photothrombotic injury. Brain sections were stained with Hoechst solution for nuclear staining (pseudo-colored green). Scale bar: 500 µm (upper panel), 200 µm (magnified images) (C) Evans blue saline solution was injected at 21 days after photothrombotic injury. Mice were perfused after circulation for 2 h, and the brains were extracted. The brain section shows that Evans blue dye was restricted within the lesion core without dispersion across the scar tissue. Scale bar: 1 mm (D) Mouse brains were extracted on day 21 after injury, and the brain sections were stained with antibodies for CD31 (the marker for endothelial cell). Vessels were rarely observed in the lesion core where the dye accumulated. Scale bar: 500 µm [N: normal tissue, LC: lesion core, FS: fibrotic scar)]. (TIF) [file pone.0094695.s001.tif]

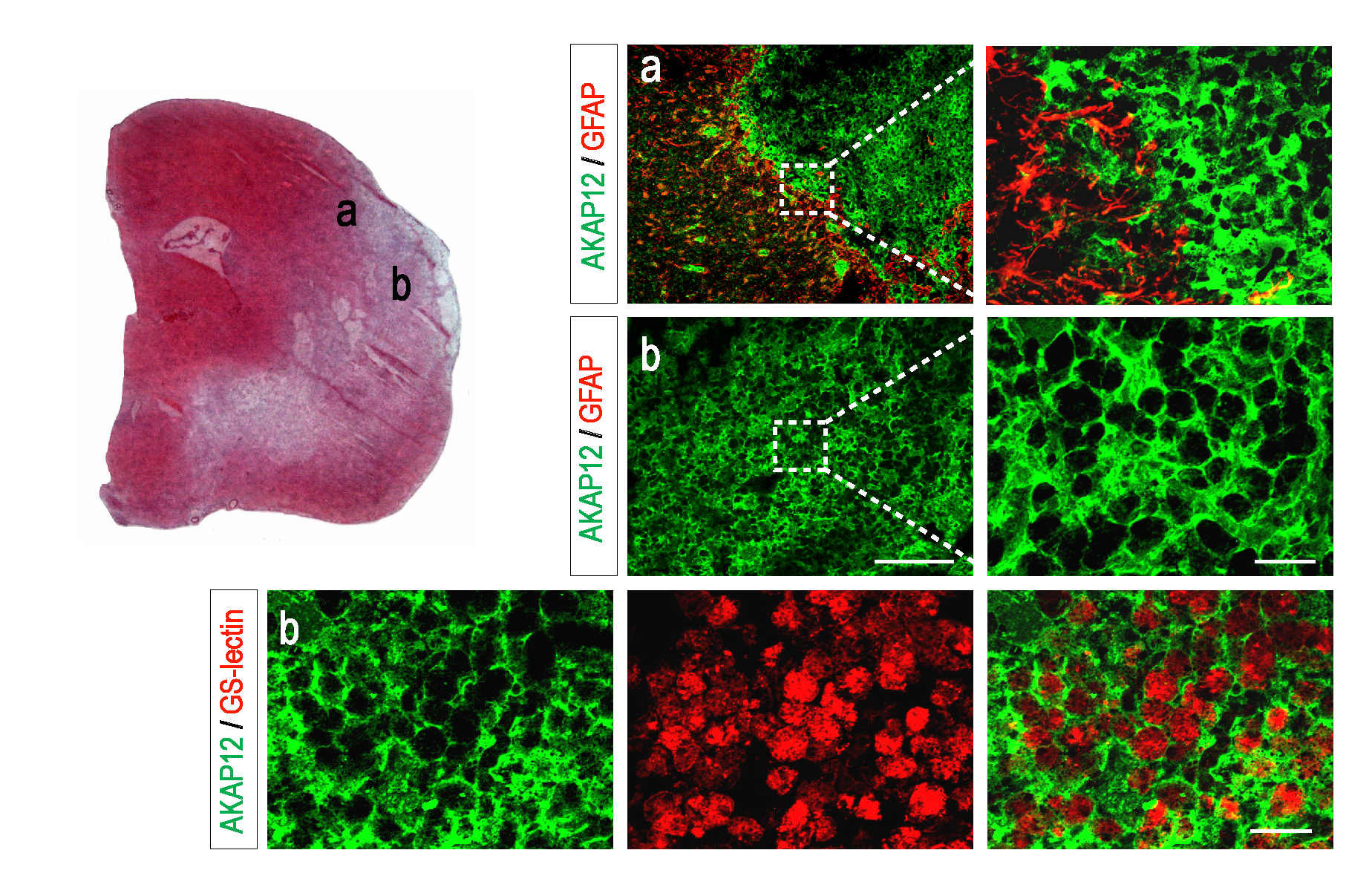

Supplement: Figure S2 — AKAP12-positive cells formed immune cell trapping structures in rodent middle cerebral artery occlusion (MCAO) injury models. The structure formed by AKAP12-positive cells is also observed in rodent middle cerebral artery occlusion (MCAO) injury models. Rat brains were harvested on day 21 after MCAO injury. [a] is the boundary of the lesion site and [b] is the fibrotic scar. Scale bar: 200 µm (left panel), 40 µm (magnified right panel), 40 µm (lower panel). (TIF) [file pone.0094695.s002.tif]

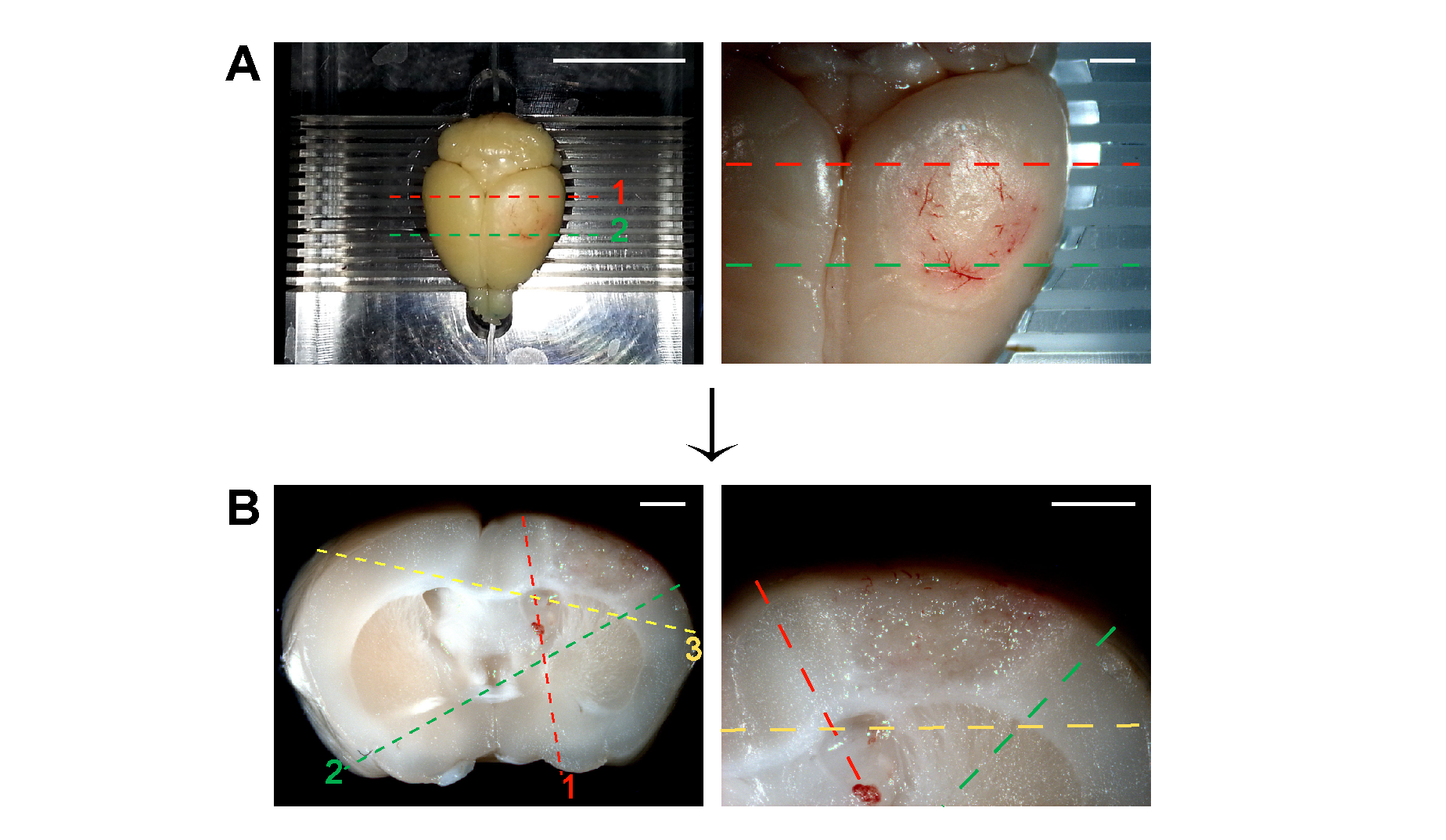

Supplement: Figure S3 — The method to define and collect the lesion tissue. (A) After perfusion, the lesion site was easily distinguishable from the normal tissue. Injured mouse brains were coronal-dissected at the inside from the lesion boundary with a razor blade in a mold. Scale bar: 1 cm, 1 mm (magnified image) (B) Since the scar tissue is white compared to adjacent tissues due to its high cell density, the scar tissue becomes the border line between the normal and the lesion tissue. To harvest both the lesion and scar tissue, brain coronal sections were dissected with a razor blade in three directions along the outside of the scar tissue. Scale bar: 1 mm. (TIF) [file pone.0094695.s003.tif]

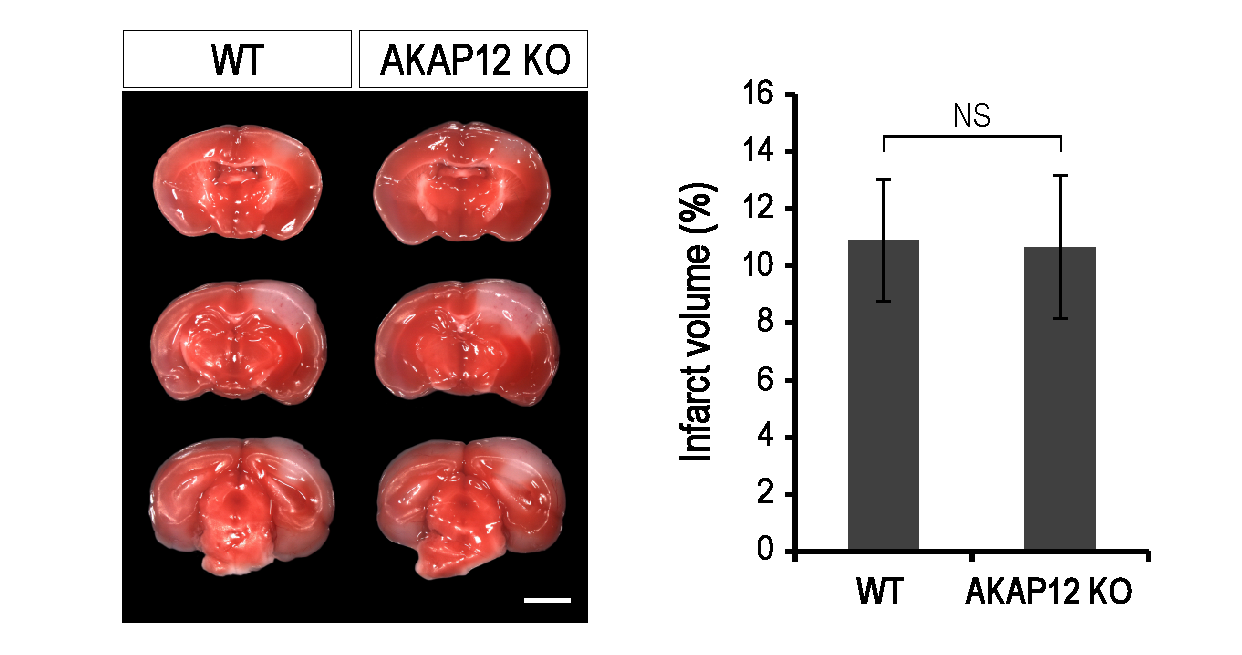

Supplement: Figure S4 — AKAP12 KO did not affect to the penumbra. Mouse brains were extracted at 1 day after injury, and serial brain slices (2 mm thickness) near the lesion site were incubated for 30 min at 37°C in 0.05% TTC solution. Pictures of the brain slices were taken after fixation with 4% PFA and analyzed using Image J program. (Mean ± S.D.; n = 4 mice per WT and KO; an unpaired two-tailed Student t-test: NS: not significant) Scale bar: 2 mm. (TIF) [file pone.0094695.s004.tif]
